# 1-(4-Chloro­phen­yl)-3-(3,4-dimethyl­phen­yl)prop-2-en-1-one

**DOI:** 10.1107/S1600536810031880

**Published:** 2010-08-18

**Authors:** Meng Guo

**Affiliations:** a Microscale Science Institute, Weifang University, Weifang 261061, People’s Republic of China

## Abstract

The title compound, C_17_H_15_ClO, was prepared from 3,4-dimethyl­benzaldehyde and 4-chloro­hypnone by Aldol condensation. The dihedral angle formed by the two benzene rings is 48.91 (8)°. Only van der Waals forces affect the packing.

## Related literature

For background to the aplications of chalcones, see: Anto *et al.* (1994 ▶); Hsieh *et al.* (1998 ▶). For a related structure, see: Zhou (2010 ▶). 
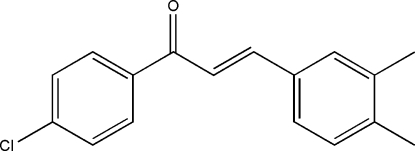


## Experimental

### 

#### Crystal data

C_17_H_15_ClO
*M* *_r_* = 270.74
Triclinic, 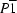

*a* = 5.9621 (12) Å
*b* = 7.7369 (15) Å
*c* = 15.513 (3) Å
α = 98.30 (3)°
β = 99.96 (3)°
γ = 95.23 (3)°
*V* = 692.5 (2) Å^3^
*Z* = 2
Mo *K*α radiation
μ = 0.26 mm^−1^
*T* = 293 K
0.25 × 0.20 × 0.18 mm

#### Data collection

Bruker SMART CCD diffractometer
6689 measured reflections
3141 independent reflections
2643 reflections with *I* > 2σ(*I*)
*R*_int_ = 0.030

#### Refinement

*R*[*F* ^2^ > 2σ(*F* ^2^)] = 0.061
*wR*(*F* ^2^) = 0.188
*S* = 1.08
3141 reflections
172 parameters
H-atom parameters constrained
Δρ_max_ = 0.54 e Å^−3^
Δρ_min_ = −0.52 e Å^−3^

### 

Data collection: *SMART* (Bruker, 1997 ▶); cell refinement: *SAINT* (Bruker, 1997 ▶); data reduction: *SAINT*; program(s) used to solve structure: *SHELXS97* (Sheldrick, 2008 ▶); program(s) used to refine structure: *SHELXL97* (Sheldrick, 2008 ▶); molecular graphics: *SHELXTL* (Sheldrick, 2008 ▶); software used to prepare material for publication: *SHELXTL*.

## Supplementary Material

Crystal structure: contains datablocks global, I. DOI: 10.1107/S1600536810031880/hb5576sup1.cif

Structure factors: contains datablocks I. DOI: 10.1107/S1600536810031880/hb5576Isup2.hkl

Additional supplementary materials:  crystallographic information; 3D view; checkCIF report

## Figures and Tables

**Fig. 1.**
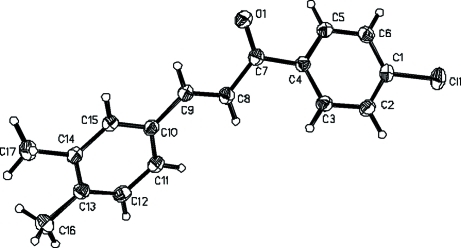
The molecular structure of (I) with displacement ellipsoids drawn at the 30% probability level.

## supplementary crystallographic information

### Comment

Among flavonoids, chalcones have been identified as interesting compounds having multiple biological actions which include antiinflammatory (Hsieh *et al.*,1998) and antioxidant (Anto *et al.*,1994). As part of our search for new biologically active compounds we synthesized the title compound (I) and report its crystal structure herein.

In the crystal structure of compound(I)(Fig.1),the dihedral angle between the two benzene rings(C1—C6) and (C10—C15) is 48.91 (8)°. All of the bond lengths and bond angles are in normal ranges and comparable to those in a related structure (Zhou,2010).

### Experimental

A mixture of the 4-chlorohypnone (0.01 mol) and 3,4-dimethylbenzaldehyde(0.01 mol) and 10% NaOH (10 ml) was stirred in ethanol (30 ml) for 3 h to afford the title compound (yield 65%). Yellow bars of (I) were obtailed by recrystallization from ethyl acetate at room temperature.

### Refinement

H atoms were positioned geometrically and allowed to ride on their parent atoms, with C—H distances of 0.93–0.96 Å, and with *U*_iso_(H) = 1.2-1.5*U*_eq_ of the parent atoms.

### Crystal data

**Table:**

C_17_H_15_ClO	*Z* = 2
*M**_r_* = 270.74	*F*(000) = 284
Triclinic, *P*1	*D*_x_ = 1.299 Mg m^−^^3^
Hall symbol: -P 1	Mo *K*α radiation, λ = 0.71073 Å
*a* = 5.9621 (12) Å	Cell parameters from 2643 reflections
*b* = 7.7369 (15) Å	θ = 3.2–27.5°
*c* = 15.513 (3) Å	µ = 0.26 mm^−^^1^
α = 98.30 (3)°	*T* = 293 K
β = 99.96 (3)°	Bar, yellow
γ = 95.23 (3)°	0.25 × 0.20 × 0.18 mm
*V* = 692.5 (2) Å^3^	

### Data collection

**Table:**

Bruker SMART CCD diffractometer	2643 reflections with *I* > 2σ(*I*)
Radiation source: fine-focus sealed tube	*R*_int_ = 0.030
graphite	θ_max_ = 27.5°, θ_min_ = 3.2°
phi and ω scans	*h* = −7→7
6689 measured reflections	*k* = −10→10
3141 independent reflections	*l* = −20→18

### Refinement

**Table:**

Refinement on *F*^2^	Primary atom site location: structure-invariant direct methods
Least-squares matrix: full	Secondary atom site location: difference Fourier map
*R*[*F*^2^ > 2σ(*F*^2^)] = 0.061	Hydrogen site location: inferred from neighbouring sites
*wR*(*F*^2^) = 0.188	H-atom parameters constrained
*S* = 1.08	*w* = 1/[σ^2^(*F*_o_^2^) + (0.1287*P*)^2^ + 0.0873*P*] where *P* = (*F*_o_^2^ + 2*F*_c_^2^)/3
3141 reflections	(Δ/σ)_max_ < 0.001
172 parameters	Δρ_max_ = 0.54 e Å^−^^3^
0 restraints	Δρ_min_ = −0.52 e Å^−^^3^

### Special details

**Table:**

Geometry. All e.s.d.'s (except the e.s.d. in the dihedral angle between two l.s. planes) are estimated using the full covariance matrix. The cell e.s.d.'s are taken into account individually in the estimation of e.s.d.'s in distances, angles and torsion angles; correlations between e.s.d.'s in cell parameters are only used when they are defined by crystal symmetry. An approximate (isotropic) treatment of cell e.s.d.'s is used for estimating e.s.d.'s involving l.s. planes.
Refinement. Refinement of *F*^2^ against ALL reflections. The weighted *R*-factor *wR* and goodness of fit *S* are based on *F*^2^, conventional *R*-factors *R* are based on *F*, with *F* set to zero for negative *F*^2^. The threshold expression of *F*^2^ > σ(*F*^2^) is used only for calculating *R*-factors(gt) *etc*. and is not relevant to the choice of reflections for refinement. *R*-factors based on *F*^2^ are statistically about twice as large as those based on *F*, and *R*- factors based on ALL data will be even larger.

### Fractional atomic coordinates and isotropic or equivalent isotropic displacement parameters (Å^2^)

**Table:**

	*x*	*y*	*z*	*U*_iso_*/*U*_eq_	
Cl1	0.06653 (10)	0.43200 (7)	−0.40023 (3)	0.0645 (2)	
C2	0.2286 (3)	0.4248 (2)	−0.22733 (12)	0.0442 (4)	
H2A	0.3675	0.4759	−0.2370	0.053*	
C15	0.3097 (3)	0.2253 (2)	0.26373 (11)	0.0398 (4)	
H15A	0.1822	0.2763	0.2774	0.048*	
C10	0.3326 (3)	0.1916 (2)	0.17512 (11)	0.0372 (4)	
C1	0.0394 (3)	0.3865 (2)	−0.29529 (11)	0.0419 (4)	
C9	0.1543 (3)	0.2354 (2)	0.10668 (11)	0.0405 (4)	
H9A	0.0194	0.2644	0.1245	0.049*	
C7	−0.0361 (3)	0.2764 (2)	−0.04009 (11)	0.0417 (4)	
C6	−0.1707 (3)	0.3131 (2)	−0.28266 (12)	0.0476 (4)	
H6A	−0.2959	0.2877	−0.3293	0.057*	
O1	−0.2265 (2)	0.2775 (2)	−0.02091 (9)	0.0582 (4)	
C11	0.5261 (3)	0.1151 (2)	0.15544 (12)	0.0425 (4)	
H11A	0.5490	0.0942	0.0972	0.051*	
C8	0.1647 (3)	0.2382 (2)	0.02181 (12)	0.0449 (4)	
H8A	0.2987	0.2161	0.0013	0.054*	
C4	−0.0022 (3)	0.3133 (2)	−0.12946 (10)	0.0369 (4)	
C3	0.2072 (3)	0.3857 (2)	−0.14451 (11)	0.0425 (4)	
H3A	0.3340	0.4081	−0.0985	0.051*	
C12	0.6825 (3)	0.0710 (2)	0.22314 (12)	0.0445 (4)	
H12A	0.8084	0.0181	0.2092	0.053*	
C14	0.4693 (3)	0.1859 (2)	0.33241 (11)	0.0412 (4)	
C13	0.6579 (3)	0.1031 (2)	0.31155 (12)	0.0417 (4)	
C5	−0.1902 (3)	0.2784 (2)	−0.19930 (12)	0.0441 (4)	
H5A	−0.3309	0.2311	−0.1895	0.053*	
C17	0.4398 (4)	0.2300 (4)	0.42722 (13)	0.0626 (6)	
H17A	0.5647	0.1938	0.4657	0.094*	
H17B	0.2977	0.1697	0.4343	0.094*	
H17C	0.4381	0.3546	0.4422	0.094*	
C16	0.8291 (4)	0.0483 (3)	0.38260 (15)	0.0594 (5)	
H16A	0.9448	−0.0063	0.3562	0.089*	
H16B	0.7525	−0.0338	0.4119	0.089*	
H16C	0.8994	0.1499	0.4250	0.089*	

### Atomic displacement parameters (Å^2^)

**Table:**

	*U*^11^	*U*^22^	*U*^33^	*U*^12^	*U*^13^	*U*^23^
Cl1	0.0855 (4)	0.0709 (4)	0.0412 (3)	0.0036 (3)	0.0171 (2)	0.0203 (2)
C2	0.0418 (8)	0.0477 (9)	0.0442 (9)	−0.0002 (7)	0.0126 (7)	0.0093 (7)
C15	0.0371 (7)	0.0445 (8)	0.0399 (8)	0.0054 (6)	0.0118 (6)	0.0086 (7)
C10	0.0372 (7)	0.0368 (7)	0.0384 (8)	0.0012 (6)	0.0094 (6)	0.0085 (6)
C1	0.0527 (9)	0.0393 (8)	0.0353 (8)	0.0056 (7)	0.0108 (7)	0.0087 (6)
C9	0.0418 (8)	0.0423 (8)	0.0391 (8)	0.0037 (6)	0.0106 (6)	0.0094 (6)
C7	0.0433 (8)	0.0462 (8)	0.0369 (8)	0.0062 (6)	0.0105 (6)	0.0067 (7)
C6	0.0450 (8)	0.0537 (10)	0.0407 (9)	0.0009 (7)	−0.0002 (7)	0.0092 (7)
O1	0.0451 (7)	0.0882 (10)	0.0464 (7)	0.0088 (6)	0.0163 (5)	0.0177 (7)
C11	0.0449 (8)	0.0433 (8)	0.0411 (9)	0.0041 (7)	0.0150 (7)	0.0052 (7)
C8	0.0457 (8)	0.0541 (10)	0.0383 (8)	0.0103 (7)	0.0119 (7)	0.0116 (7)
C4	0.0385 (7)	0.0377 (7)	0.0353 (8)	0.0056 (6)	0.0084 (6)	0.0058 (6)
C3	0.0371 (7)	0.0493 (9)	0.0390 (8)	0.0010 (6)	0.0049 (6)	0.0053 (7)
C12	0.0410 (8)	0.0414 (8)	0.0527 (10)	0.0064 (6)	0.0139 (7)	0.0056 (7)
C14	0.0397 (8)	0.0463 (8)	0.0387 (8)	0.0012 (6)	0.0108 (6)	0.0090 (7)
C13	0.0384 (8)	0.0386 (8)	0.0475 (9)	0.0010 (6)	0.0058 (7)	0.0102 (7)
C5	0.0374 (8)	0.0494 (9)	0.0443 (9)	−0.0002 (7)	0.0058 (7)	0.0093 (7)
C17	0.0549 (10)	0.0958 (16)	0.0399 (9)	0.0154 (10)	0.0116 (8)	0.0130 (10)
C16	0.0561 (10)	0.0655 (12)	0.0585 (12)	0.0147 (9)	0.0040 (9)	0.0194 (10)

### Geometric parameters (Å, °)

**Table:**

Cl1—C1	1.7455 (17)	C11—H11A	0.9300
C2—C1	1.382 (3)	C8—H8A	0.9300
C2—C3	1.386 (3)	C4—C3	1.392 (2)
C2—H2A	0.9300	C4—C5	1.394 (2)
C15—C14	1.389 (2)	C3—H3A	0.9300
C15—C10	1.394 (2)	C12—C13	1.394 (2)
C15—H15A	0.9300	C12—H12A	0.9300
C10—C11	1.403 (2)	C14—C13	1.406 (2)
C10—C9	1.467 (2)	C14—C17	1.505 (3)
C1—C6	1.385 (3)	C13—C16	1.504 (2)
C9—C8	1.332 (2)	C5—H5A	0.9300
C9—H9A	0.9300	C17—H17A	0.9600
C7—O1	1.222 (2)	C17—H17B	0.9600
C7—C8	1.481 (2)	C17—H17C	0.9600
C7—C4	1.499 (2)	C16—H16A	0.9600
C6—C5	1.380 (3)	C16—H16B	0.9600
C6—H6A	0.9300	C16—H16C	0.9600
C11—C12	1.383 (3)		
			
C1—C2—C3	118.67 (15)	C5—C4—C7	118.34 (15)
C1—C2—H2A	120.7	C2—C3—C4	120.69 (15)
C3—C2—H2A	120.7	C2—C3—H3A	119.7
C14—C15—C10	122.73 (15)	C4—C3—H3A	119.7
C14—C15—H15A	118.6	C11—C12—C13	122.25 (15)
C10—C15—H15A	118.6	C11—C12—H12A	118.9
C15—C10—C11	118.03 (15)	C13—C12—H12A	118.9
C15—C10—C9	119.14 (14)	C15—C14—C13	118.70 (15)
C11—C10—C9	122.83 (15)	C15—C14—C17	120.56 (15)
C2—C1—C6	122.03 (16)	C13—C14—C17	120.74 (16)
C2—C1—Cl1	118.89 (13)	C12—C13—C14	118.63 (15)
C6—C1—Cl1	119.08 (13)	C12—C13—C16	120.30 (16)
C8—C9—C10	127.04 (15)	C14—C13—C16	121.07 (16)
C8—C9—H9A	116.5	C6—C5—C4	121.01 (16)
C10—C9—H9A	116.5	C6—C5—H5A	119.5
O1—C7—C8	122.36 (16)	C4—C5—H5A	119.5
O1—C7—C4	119.56 (16)	C14—C17—H17A	109.5
C8—C7—C4	118.08 (14)	C14—C17—H17B	109.5
C5—C6—C1	118.51 (15)	H17A—C17—H17B	109.5
C5—C6—H6A	120.7	C14—C17—H17C	109.5
C1—C6—H6A	120.7	H17A—C17—H17C	109.5
C12—C11—C10	119.58 (15)	H17B—C17—H17C	109.5
C12—C11—H11A	120.2	C13—C16—H16A	109.5
C10—C11—H11A	120.2	C13—C16—H16B	109.5
C9—C8—C7	120.28 (16)	H16A—C16—H16B	109.5
C9—C8—H8A	119.9	C13—C16—H16C	109.5
C7—C8—H8A	119.9	H16A—C16—H16C	109.5
C3—C4—C5	119.06 (15)	H16B—C16—H16C	109.5
C3—C4—C7	122.57 (14)		

## References

[bb1] Anto, R. J., Kuttan, G., Kuttan, R., Sathyanarayana, K. & Rao, M. N. A. (1994). *J. Clin. Biochem. Nutr.* **17**, 73–80.

[bb2] Bruker (1997). *SMART* and *SAINT* Bruker AXS Inc., Madison, Wisconsin, USA.

[bb3] Hsieh, H. K., Lee, T. H., Wang, J. P., Wang, J. J. & Lin, C. N. (1998). *Pharm. Res.* **15**, 39–46. 10.1023/a:1011940401754 9487544

[bb4] Sheldrick, G. M. (2008). *Acta Cryst.* A**64**, 112–122. 10.1107/S0108767307043930 18156677

[bb5] Zhou, Y. (2010). *Acta Cryst.* E**66**, o1412. 10.1107/S1600536810018106 PMC2979457 21579491

